# The dual-inhibitory effect of miR-338-5p on the multidrug resistance and cell growth of hepatocellular carcinoma

**DOI:** 10.1038/s41392-017-0003-4

**Published:** 2018-01-26

**Authors:** Yang Zhao, Jing Chen, Wenxin Wei, Xinming Qi, Chunzhu Li, Jin Ren

**Affiliations:** 1 Center for Drug Safety Evaluation and Research, State Key Laboratory of Drug Research, Shanghai Institute of Materia Medica, Chinese Academy of Sciences, Shanghai, 201203 China; 2 Center for Drug Safety Evaluation and Research, State Key Laboratory of Drug Research, Shanghai Institute of Materia Medica, University of Chinese Academy of Sciences, Beijing, 100049 China; 3 Department of Hepatic Surgery, Eastern Hepatobiliary Surgery Hospital, Second Military Medical University, Shanghai, 200438 China

## Abstract

Chemotherapeutic treatments against hepatocellular carcinoma (HCC) are necessary for both inoperable patients to improve prospects for survival and surgery patients to improve the outcome after surgical resection. However, multidrug resistance (MDR) is a major obstacle to obtaining desirable results. Currently, increasing the chemotherapy sensitivity of tumor cells or discovering novel tumor inhibitors is an effective therapeutic strategy to solve this issue. In the present study, we uncovered the dual-inhibitory effect of miR-338-5p: on the one hand, it could downregulate ABCB1 expression and sensitize HCC cells to doxorubicin and vinblastine by directly targeting the 3′-untranslated region (3′-UTR) of ABCB1, while, on the other hand, it could suppress the proliferation of HCC cells by directly targeting the 3′-UTR of EGFR and reducing EGFR expression. Since EGFR regulates ABCB1 levels, the indirect action of miR-338-5p in ABCB1 modulation was revealed, in which miR-338-5p inhibits ABCB1 expression by targeting the EGFR/ERK1/2 signaling pathway. These data indicate that the miR-338-5p/EGFR/ABCB1 regulatory loop plays a critical role in HCC, and a negative correlation between miR-338-5p and EGFR or ABCB1 was also detected in HCC clinical samples. In conclusion, these findings reveal a critical role for miR-338-5p in the regulation of MDR and proliferation of HCC, suggesting the potential therapeutic implications of miR-338-5p in HCC treatment.

## Liver cancer: Therapeutic potential of a microRNA

A small RNA molecule inhibits the growth of liver cancer cells while also making the cells sensitive to the anti-cancer drugs. These twin effects of the natural microRNA miR-338-5p were discovered by researchers in China, led by Chunzhu Li and Jin Ren at the Center for Drug Safety Evaluation and Research in Shanghai. MicroRNAs control gene activity by interacting with the messenger RNA copies of genes that guide synthesis of the proteins the genes encode. The research identified a gene whose expression miR-338-5p inhibits to restrict the growth of hepatocellular carcinoma – the most common form of liver cancer. This is also one of the most drug-resistant forms of liver cancer. A different gene whose activity miR-338-5p controls to sensitize cells to chemotherapeutic drugs was also identified. Using miR-338-5p to treat liver cancer warrants further investigation.

## Introduction

Hepatocellular carcinoma (HCC) is one of the most prevalent forms of cancer and a leading cause of cancer mortality globally.^1,2^ The frontline treatment for this disease is orthotopic liver transplantation and hepatic resection.^3,4^ Unfortunately, most of the tumors are at advanced stages and often inoperable when diagnosed.^5^ Chemotherapy is commonly used as a primary treatment in inoperable patients or as an adjuvant therapy after surgical resection. However, the successful treatment of HCC with chemotherapeutic agents is often hampered by the multidrug resistance (MDR) of this cancer.^6^ MDR can be caused by the high expression of adenosine triphosphate (ATP)-binding cassette (ABC) transporter family members, which mediate the ATP-dependent efflux of chemotherapeutic drugs out of cancer cells.^7^

So far, developing inhibitors of MDR-related proteins is one of the solutions for MDR in cancers. Based on the important role of ABC transporters in the MDR of cancers, numerous inhibitors of ABC transporters were discovered to improve the efficacy of anticancer agents in resistant tumor tissues in recent decades.^8,9^ However, most of these drugs failed in the clinic because of undesired side effects and toxicity issues.^10,11^ In addition, identifying novel anti-tumor candidates with low probability to develop resistance in cancers may also be another feasible strategy to avoid MDR. Thus, exploring new chemosensitizers that inhibit the activities of MDR-related genes or developing new anti-cancer candidates that suppress tumor cell replication may be crucial for the successful treatment of HCC.

MicroRNAs (miRNAs) are small endogenous non-coding RNAs with 18–25 nucleotides that can trigger either mRNA translational suppression or mRNA degradation.^12^ Multiple miRNAs are capable of modulating MDR in cancers.^13,14^ Zhu et al.^15^ found that miR-181b modulated multidrug resistance by targeting BCL2 in human cancer cell lines. miR-133a and miR-326 sensitized HepG2 cells to ADM (adriamycin) through modulating ABCC1 expression.^16^ In addition to the regulation of MDR, recent studies have shown that specific miRNAs could contribute to cell proliferation and metastasis in cancers.^17,18^ For instance, miR-32 promoted growth, migration and invasion in colorectal carcinoma cells.^19^ Lal et al.^20^ found that miR-24 inhibits cell proliferation by targeting E2F2, MYC, and other cell cycle genes via binding to “seedless” 3′-UTR microRNA recognition elements. Therefore, miRNA targeting of MDR- or proliferation-associated genes has been demonstrated as effective in cancer therapy.

In the present study, we found that miR-338-5p could suppress the expression of ABCB1, a drug transporter and the main reason for MDR, and sensitize HCC cells to doxorubicin (DOX) and vinblastine (VBL), two chemotherapeutic drugs and P-gp (P-glycoprotein) substrates. We further observed that miR-338-5p could inhibit the proliferation of HCC cells by directly targeting EGFR. Moreover, these results suggested that miR-338-5p downregulated ABCB1 via a dual inhibitory pathway. In addition to directly interacting with the ABCB1 3′-UTR, miR-338-5p could target the EGFR/ERK1/2 pathway to inhibit the expression of ABCB1, resulting in increased sensitivity of hepatoma cells to DOX. These results indicated that miR-338-5p might be a new potential therapeutic target for HCC treatment.

## Materials and methods

### Cell culture and tissue samples

Two human hepatocellular carcinoma cell lines, Hep3B and Huh7^21,22^, were obtained from the Shanghai Institutes for Biological Sciences, Chinese Academy of Sciences. Cells were cultured in DMEM (HyClone, #AC10232473, USA) supplemented with 10% fetal bovine serum (FBS) (Sigma, #F2442, USA) and antibiotics (antibiotic-antimycotic, 50 units mL^−1^ each) (Invitrogen, #15240062). All cells were cultured at 37 °C in a humidified air atmosphere at 5% CO_2_.

Human hepatocellular carcinoma tissues were obtained from Eastern Hospital of Hepatobiliary Surgery (Shanghai, China). No patients received any local or systemic anticancer treatments before the surgery. The present study was approved by the Ethics Committee of Eastern Hospital of Hepatobiliary Surgery.

### Cell transfection

miRNA mimics, inhibitors, siRNAs and their negative control oligonucleotides (NC) were purchased from GenePharm (Shanghai, China). ABCB1 and EGFR overexpression plasmids that carried no binding sites for miR-338-5p were purchased from Sino Biological Inc. (#HG12030-UT, #HG10001-UT) (Beijing, China). After seeding onto six-well plates at 2 × 10^5^ cells/well, the cells were cultured for 16 h and then transfected with the oligonucleotides or plasmids using Lipofectamine 2000 reagent and OPTI-MEM I reduced serum medium (Invitrogen, #1836503) according to the manufacturer’s instructions.

### Drug sensitivity assay

At densities of 2 × 10^5^/well, the cells were cultured in six-well plates for 16 h and then transfected with 50 nM miR-338-5p/NC mimics or inhibitor. Next, 5 × 10^3^ cells were reseeded onto 96-well plates at 72 h after transfection and treated with medium containing DOX (Aladdin, #g1509018) or VBL (J&K, #9C0L18) for 48 h. Finally, 10 μL of CCK-8 (Cell Counting Kit-8, DojinDo, #kp774) was added to each well and incubated for a further 2 h. The absorbance of each sample was measured at 450 nm by the microplate reader.

### Flow cytometry

The fluorescence intensity of intracellular DOX was measured by flow cytometry. The cells were seeded onto six-well plates and cultured overnight at 37 °C. Then, the cells were transfected with 50 nM miR-338-5p/NC mimics or inhibitors. After 72 h, the cells were incubated in medium containing DOX (15 μM) (CALBIOCHEM, #324380) for 3 h. At the end of incubation, the cells were washed thrice with cold PBS, and the DOX fluorescence intensity in the cells was measured using a FACSCalibur flow cytometer (BD Biosciences, Franklin Lakes, NJ, USA).

### Cell proliferation analysis

Hep3B and Huh7 cells were seeded onto 96-well/six-well plates and transfected with small RNAs (50 nM miR-338-5p mimics, inhibitor or NC) or plasmids (vector, ABCB1 or EGFR expression construct). At 72, 96 and 120 h after transfection, the cells were incubated with CCK-8 for 2 h, and subsequently, the absorbance was measured at 450 nm.

### Colony formation assays

A total of 3000 Hep3B or Huh7 cells per well were seeded onto a six-well plate and transfected with small RNAs (50 nM miR-338-5p mimics, inhibitor or NC) or plasmids (vector, ABCB1 or EGFR expression construct). After 14 days, cells were washed twice with cold PBS, fixed with methyl alcohol, stained by Giemsa and photographed using a Typhoon FLA 9500 instrument (GE Healthcare, Little Chalfont, UK). The colony numbers were counted using ImageQuant TL (GE Healthcare, Little Chalfont, UK).

### RNA isolation and real-time qRT–PCR

Total RNA was extracted from cells or tissues by using the UNIQ-10/Trizol total RNA extraction kit (Sangon, #B511361, Shanghai, China) and converted to cDNA using the PrimeScript RT Reagent Kit (Takara, #a4302-1, Otus, Shiga, Japan). The SYBR Green PCR kit (Takara, #aka505, Otus, Shiga, Japan) was used for qRT–PCR. The expression levels of gene mRNA were normalized to the GAPDH levels. The following primer sequences were used: ABCB1 (human) sense 5′-CCCATCATTGCAATAGCAGG-3′ and antisense 5′-TGTTCAAACTTCTGCTCCTGA-3′, EGFR (human) sense 5′-TCTACAACCCCACCACGTAC-3′ and antisense 5′-TTCCGTTACACACTTTGCGG-3′, IL6 (human) sense 5′-AGTCCTGATCCAGTTCCTGC-3′ and antisense 5′-AAGCTGCGCAGAATGAGATG-3′, IL16 (human) sense 5′-AAAACATTTTGCGCGCACAA-3′ and antisense 5′-ACCCAGGCACATCATCAGAA-3′, and GAPDH (human) sense 5′-GGTGGTCTCCTCTGACTTCAACA-3′ and antisense 5′-GTTGCTGTAGCCAAATTCGTTGT-3′.

miRNA was isolated using the *mir*Vana^TM^ miRNA isolation kit (Ambion, #AM1556, Austin, TX). MicroRNA cDNAs were synthesized using the Taqman® MicroRNA Reverse Transcription Kit (Invitrogen, #4366596). Taqman® MiRNA Assays (Invitrogen, #4427975) were used to amplify the expression of miR-338-5p (ID 002658) and RNU6B (ID 001093) according to the manufacturer’s instructions. The amplification and detection of specific products were performed with the Rotor-Gene Q 2plex HRM System (Qiagen, Valencia, CA, USA).

### Western blot analysis

Tissue or cell protein was solubilized in radio immunoprecipitation assay (RIPA) lysis buffer (Beyotime, #p0013c, China) containing 1:1000 phenylmethanesulfonyl fluoride (Beyotime, #st506, China). Equal quantities of proteins were separated by 8% SDS–PAGE and subsequently transferred onto PVDF membranes (Millipore, #IPVH00010, Billerica, MA, USA). Primary antibodies against P-gp (1:1000; Abcam, #170904, USA), EGFR (1:1000; Cell Signaling Technology, #4267, Danvers, MA, USA), p-EGFR (1:1000; #3777, Cell Signaling Technology, Danvers, MA, USA), ERK1/2 (1:1000; #4695, Cell Signaling Technology, Danvers, MA, USA), p-ERK1/2 (1:1000; #4370, Cell Signaling Technology, Danvers, MA, USA), and β-actin (1:1000, #sc4778, Santa Cruz Biotechnology, USA) were incubated with membranes. Then, antibody binding was assessed by incubation with HRP-conjugated secondary antibodies (Jackson Immuno Research Laboratories, #115-035-003, Inc., USA). The signals were detected using an ECL Plus immunoblotting detection system (Millipore, #WBKLS0500, Billerica, MA, USA).

### Luciferase assay

The wild-type 3′-UTR of ABCB1 or EGFR mRNA was inserted into the psiCHECK^TM^-2 Vector (Promega, #C8021, USA). Mutants lacking the miR-338-5p binding sites in the 3′-UTR of ABCB1 or EGFR was constructed using the KOD Plus Mutagenesis kit (TOYOBO, #562900, Japan) according to the manufacturer’s protocol. Seventy-two hours post transfection, the cells were lysed with 1 × passive lysis buffer, and both *Renilla* and firefly luciferase activities were measured with the Dual-Luciferase Reporter Assay System (Promega, # 0000223860, USA). Luciferase activity values were normalized to firefly luciferase values.

### Statistical analysis

Data are presented as the means ± SD of at least three independent experiments, unless otherwise mentioned. Two-tailed Student’s *t*-test or one-way analysis of variance was employed to analyze the data, unless otherwise mentioned. To analyze the relationship between the expression of miR-338-5p and its targets in HCC tumor samples, Spearman’s correlation test was used. All statistical analyses were performed using SPSS v.11.5 software (Chicago, IL, USA). A *P*-value <0.05 was considered statistically significant.

## Results

### **ABCB1 is a direct target of miR-338-5p**

**Fig. 1:**
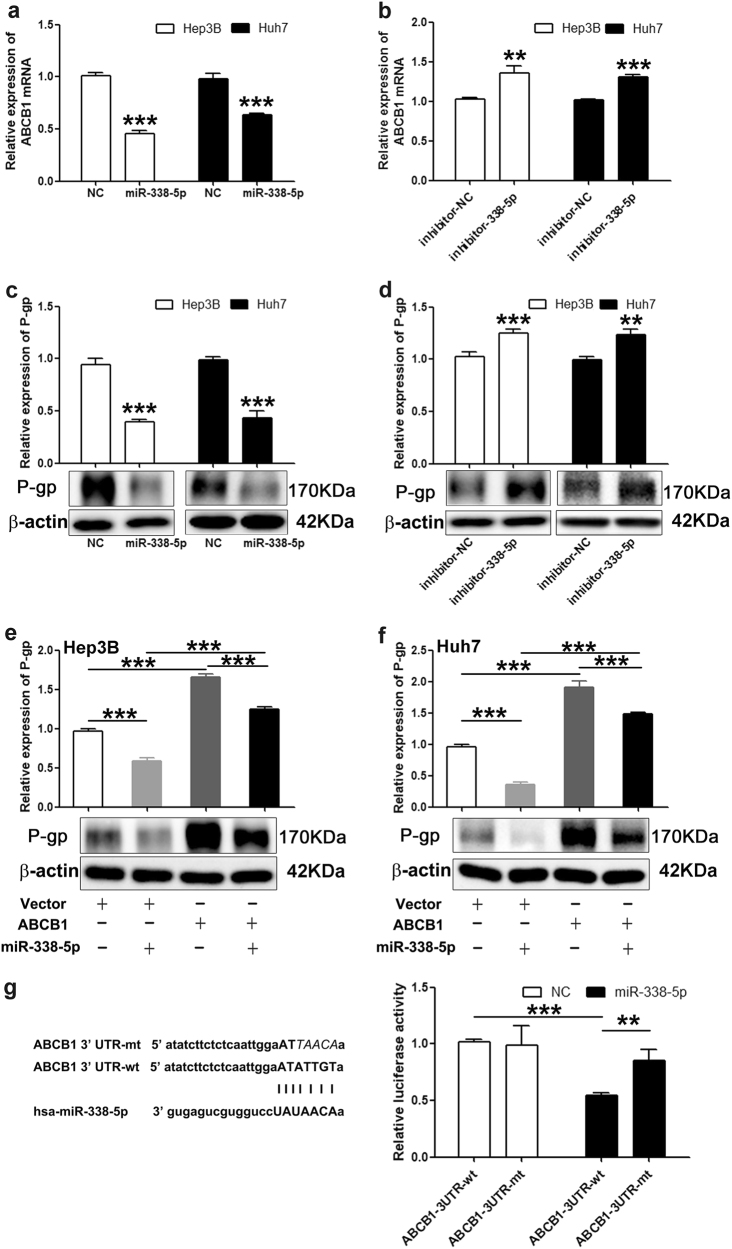
ABCB1 is a direct target of miR-338-5p. ABCB1 was detected by qRT–PCR in Hep3B and Huh7 cells treated with miR-338-5p mimics **a** or inhibitors **b**. ABCB1 mRNA levels were normalized to GAPDH mRNA levels. P-gp expression in hepatoma cells transfected with miR-338-5p mimics **c** or inhibitors **d** was analyzed by western blotting. Hep3B **e** and Huh7 **f** cells were treated with the ABCB1 vector (ABCB1, hereafter) or empty plasmid (Vector, hereafter), and then, the levels of P-gp were measured. **g** Left: alignment of miR-338-5p with ABCB1 3′-UTR. Right: luciferase assay for the direct targeting of the 3′-UTR of ABCB1 by miR-338-5p. The wide-type or mutant of ABCB1 3′-UTR plasmid was co-transfected with miR-338-5p mimics or NC in Hep3B cells, and subsequently, luciferase activity was detected. The data in all experiments are presented as the means ± SD of three independent experiments. **P < 0.01, ***P < 0.001 vs. NC or Vector

Considering the pivotal role of ABCB1, one of the key drug efflux pumps^23,24^, in MDR and the potential use of miRNA in MDR cancer therapy, we predicted candidate miRNAs that potentially bind to the 3′-UTR of human ABCB1 (P-gp) using the bioinformatics algorithms TargetScan and MicroCosm Targets in a previous study and identified the miRNAs that negatively regulated ABCB1 in hepatocarcinoma.^25^ The results of this previous study showed that miR-338-5p significantly inhibited P-gp expression and was another potential P-gp inhibitor (Figure S1). We further verified the downregulation of ABCB1 by miR-338-5p in Hep3B and Huh7 cells. The results showed that compared to negative control (NC), the expression of ABCB1 mRNA was significantly suppressed in miR-338-5p mimic-transfected cells (Fig. 1a), while the inhibition of miR-338-5p upregulated ABCB1 mRNA levels in cells (Fig. 1b). Consistently, the overexpression of miR-338-5p dramatically inhibited P-gp levels (Fig. 1c), and P-gp expression increased upon miR-338-5p inhibition in Hep3B and Huh7 cells (Fig. 1d). Furthermore, the inhibitory effects of miR-338-5p on P-gp expression in Hep3B and Huh7 cells were markedly offset by the overexpression of ABCB1 (Fig. 1e, f).

To detect whether the ABCB1 gene was the direct target of miR-338-5p, we used a luciferase reporter plasmid containing the 3′-UTR of ABCB1. Luciferase activity assays revealed that miR-338-5p overexpression decreased the luciferase activity of wild-type ABCB1 3′-UTR, and this inhibition was offset by the mutation of the target sequences in the ABCB1 3′-UTR (Fig. 1g). These results suggested that miR-338-5p downregulated ABCB1 expression via direct binding to the 3′-UTR of ABCB1.

### miR-338-5p regulates the sensitivity of HCC cells to chemotherapeutics by targeting ABCB1

**Fig. 2:**
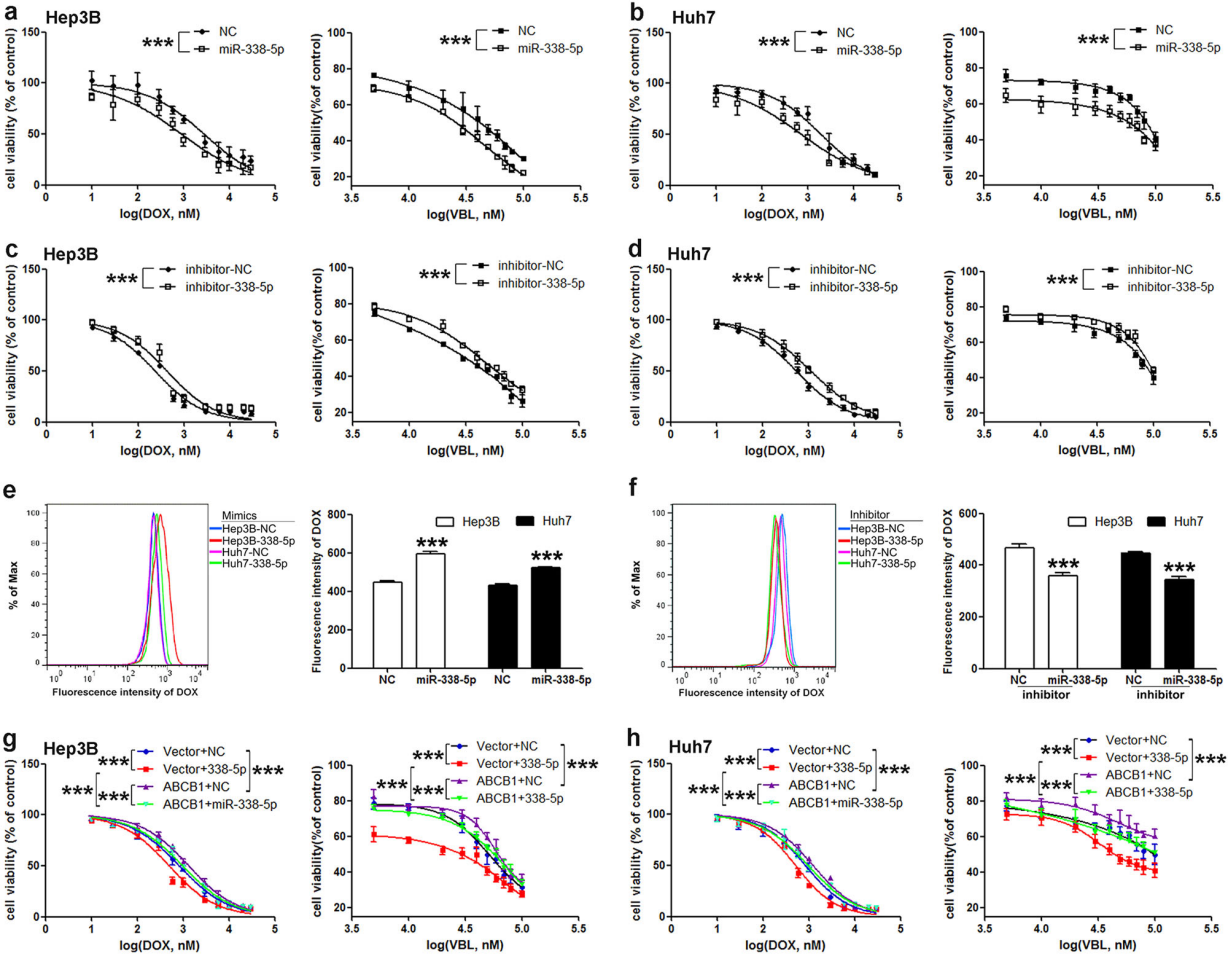
miR-338-5p regulates the sensitivity of HCC cells to DOX and VBL by targeting ABCB1. The ectopic expression of miR-338-5p enhanced the sensitivity to chemotherapeutics and increased the intracellular concentration of DOX in HCC cells. Cells were treated with medium containing DOX or VBL for 48 h, and then, cell viability was detected. Overexpression of miR-338-5p in Hep3B **a** or Huh7 **b** cells increased the sensitivity to DOX or VBL. Knockdown of miR-338-5p reduced the sensitivity to DOX or VBL of Hep3B **c** and Huh7 **d** cells. Intracellular DOX accumulation was measured by flow cytometry in cells transfected with miR-338-5p mimics **e** or inhibitors **f**. **e**–**f** Left: the output results of the intracellular DOX fluorescence intensity measured by flow cytometry. Right: the statistical results. (**g**–**h**) The sensitivity of cells to DOX or VBL was detected by CCK-8 assay. (**a**–**d**) and (**g**–**h**) Left: The sensitivity of cells to DOX. Right: The sensitivity of cells to VBL. Two-way analysis of variance was employed to analyze the drug sensitivity data. The data in all experiments are presented as the means ± SD of three independent experiments. ***P < 0.001 vs. NC

To examine the function of miR-338-5p in the MDR of HCC, miRNA mimics or inhibitors were transiently transfected into Hep3B and Huh7 cells. The results obtained from CCK-8 cell viability assays showed that the overexpression of miR-338-5p notably sensitized Hep3B or Huh7 cells to DOX or VBL compared with NC (Fig. 2a, b; Figure S2d-e). In addition, the sensitivity to DOX or VBL was decreased after miR-338-5p inhibition by an antagomir in Hep3B and Huh7 cells (Fig. 2c, d). To determine that the sensitization to chemotherapeutics was associated with the miR-338-5p regulation of P-gp function, we measured the intracellular concentration of DOX (P-gp fluorescent substrate) in cells transfected with miR-338-5p mimics or inhibitors. The results showed that the enhancement of miR-338-5p increased the intracellular DOX intensity (Fig. 2e), whereas the knockdown of miR-338-5p decreased the intracellular intensity of DOX (Fig. 2f).

To further confirm that the function of miR-338-5p in drug sensitivity was via ABCB1, the effects of overexpressing ABCB1 on cell sensitivity to DOX or VBL in the presence or absence of miR-338-5p were investigated. As shown in Fig. 2g, h, the overexpression of ABCB1 suppressed the sensitivity of miR-338-5p-overexpressing cells to DOX or VBL, which indicated that miR-338-5p regulated the sensitivity of HCC cells to DOX or VBL by targeting ABCB1. We also detected the sensitivity of ABCB1-silencing cells to DOX or VBL to validate the inhibitory effects of P-gp on drug sensitivity (Figure S2a-c).

### miR-338-5p suppresses proliferation of Hep3B and Huh7 cells

**Fig. 3:**
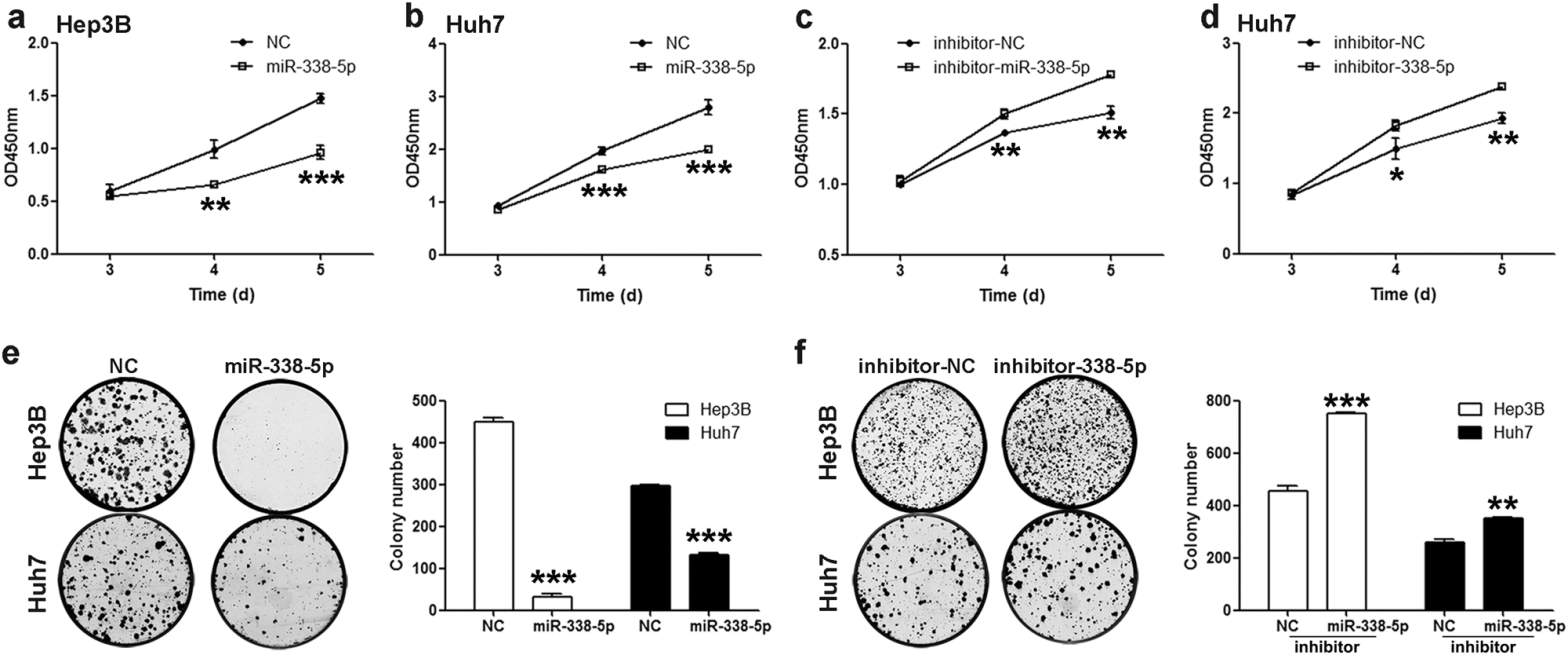
miR-338-5p suppresses proliferation of Hep3B and Huh7 cells. **a**–**b** CCK-8 assays were used to evaluate the effect of upregulation of miR-338-5p on Hep3B or Huh7 cell proliferation. **c**–**d** Cell proliferation was promoted after transfection with miR-338-5p inhibitors. **e**–**f** Cell colony formation assays showed the effects of overexpressing or knocking down miR-338-5p on HCC cell growth. Student’s *t*-test (two-tailed) was employed to analyze the data. Data in all experiments are presented as the means ± SD of three independent experiments. *P < 0.05, **P < 0.01, ***P < 0.001 vs. NC

As reported, miR-338-5p also plays different roles in the progression of several cancers.^26–28^ However, the underlying mechanism of miR-338-5p in HCC is still not well elucidated. In the present study, the cell proliferation after transfection with miR-338-5p mimics or inhibitors of Hep3B and Huh7 cells was investigated to further explore the potential functions of miR-338-5p in the tumorigenesis or tumor inhibition of HCC. The results of the 5-day cell growth assay showed that overexpression of miR-338-5p remarkably suppressed Hep3B and Huh7 cell proliferation compared with NC (Fig. 3a, b). In contrast, antagonizing endogenous miR-338-5p with its inhibitors obviously promoted Hep3B and Huh7 cell proliferation (Fig. 3c, d). Colony formation assays showed that miR-338-5p significantly inhibited colony formation in HCC cells (Fig. 3e), and its inhibition significantly promoted colony formation (Fig. 3f), which further confirmed the ability of miR-338-5p for tumor suppression. These results indicated that miR-338-5p notably suppressed human HCC cell proliferation in vitro.

### Identification of EGFR as another direct target of miR-338-5p

**Fig. 4:**
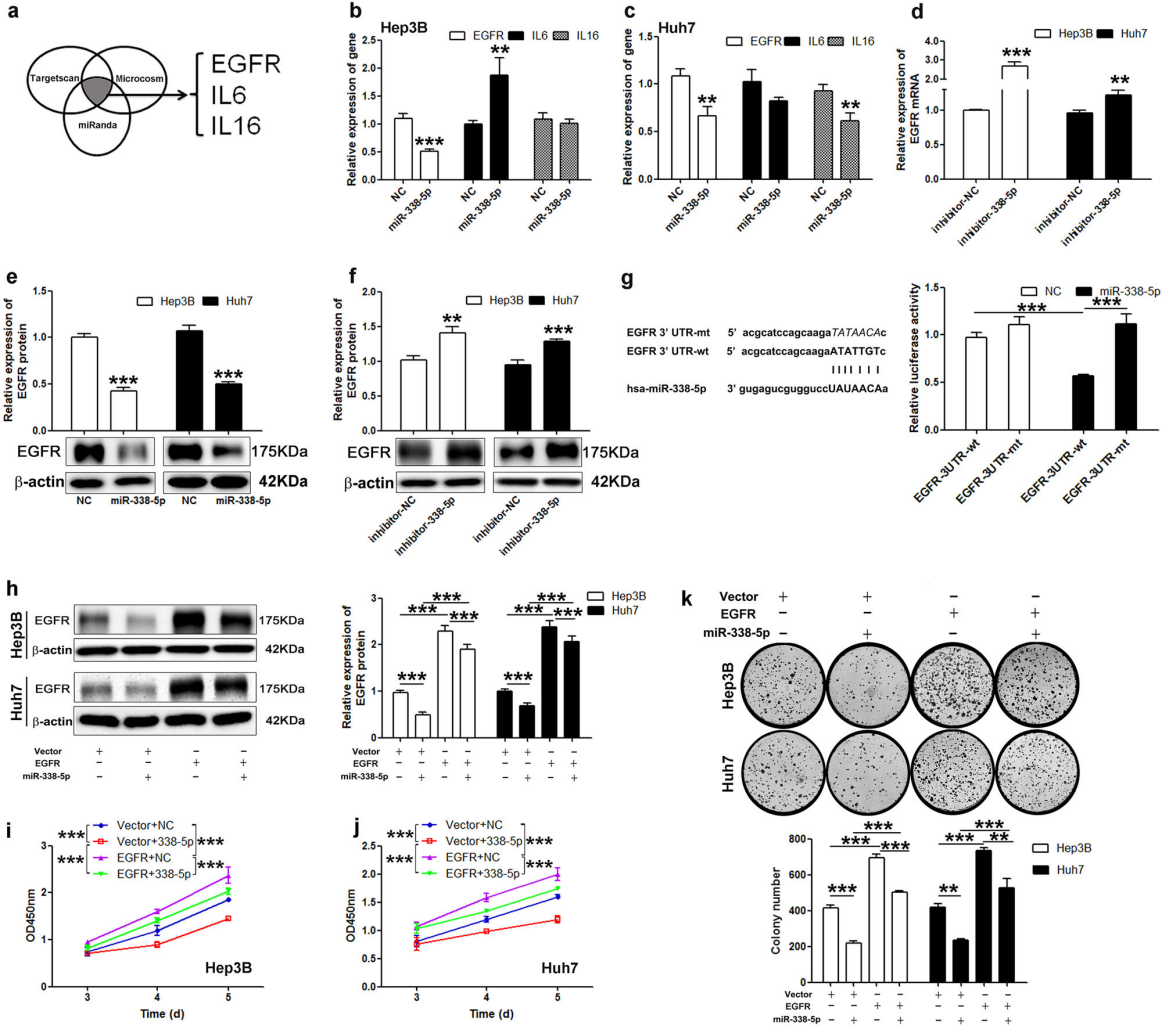
EGFR is another direct target of miR-338-5p. **a** The target genes of miR-338-5p were predicted using TargetScan, MicroCosm, and MiRanda. **b**–**c** The mRNA levels of potential target genes of miR-338-5p were measured after cells were transfected with miR-338-5p for 72 h. **d** EGFR mRNA expression was detected after cells were treated with miR-338-5p inhibitor by qRT–PCR. The protein expression of EGFR in HCC cells transfected with miR-338-5p mimics **e** or inhibitors **f** was analyzed by western blotting. **g** Left: sequence complementarity between the 3′-UTR of EGFR mRNA and the seed region of miR-338-5p. Right: luciferase activity in Hep3B cells transfected with miR-338-5p and reporter plasmids containing wt (wild-type) or mt (mutant) EGFR 3′-UTR was analyzed. **h** Cells were treated with the EGFR vector (EGFR, hereafter), and then, the protein levels of EGFR were measured by western blotting. The proliferation **i**–**j** and colony formation **k** of Hep3B and Huh7 cells were remarkably promoted after transfection with the EGFR vector compared with the control vector. Student’s *t*-test (two-tailed) or one-way or two-way analysis of variance was employed to analyze the data. Data in all experiments are presented as the means ± SD of three independent experiments. **P < 0.01, ***P < 0.001 vs. NC

Since P-gp is a direct target of miR-338-5p and silencing of P-gp in HCC cells had no effect on cell growth (Figure S2f-h), the tumor growth-related target genes of miR-338-5p should be identified to clarify the underlying mechanism of its effect on cell proliferation. First, we obtained a list of candidate target genes that might be regulated by miR-338-5p using bioinformatics algorithms, including TargetScan, MicroCosm and MiRanda. Combining the prediction results from these three algorithms, three genes were selected for further identification: EGFR, IL6 and IL16 (Fig. 4a). We detected the mRNA expression of these three genes after transfection with miR-338-5p mimics or NC in Hep3B and Huh7 cells. Interestingly, the overexpression of miR-338-5p downregulated EGFR mRNA in both Hep3B and Huh7 cells (Fig. 4b, c). In addition, the levels of EGFR mRNA were increased after treatment with miR-338-5p inhibitors in Hep3B and Huh7 cells (Fig. 4d). As shown in Fig. 4e, f, the overexpression or inhibition of miR-338-5p markedly suppressed or increased EGFR protein expression, respectively, in Hep3B and Huh7 cells compared with NC. Furthermore, luciferase activity assays showed that miR-338-5p decreased the luciferase activity of the plasmid containing the wild-type EGFR 3′-UTR but not that of the plasmid containing the mutant EGFR 3′-UTR (Fig. 4g), indicating that EGFR is a direct target of miR-338-5p.

We further validated the roles of EGFR in cell growth in HCC and investigated whether the inhibitory effects of miR-338-5p on HCC cell proliferation were mediated by EGFR. The effects of siRNA on EGFR expression were confirmed by western blot analysis (Figure S3a). Notably, silencing of EGFR inhibited the proliferation (Figure S3b and c) and colony formation (Figure S3d) of Hep3B and Huh7 cells. Furthermore, EGFR overexpression restored the inhibitory effects of miR-338-5p on EGFR expression in Hep3B and Huh7 cells (Fig. 4h). The proliferation (Fig. 4i, j) and colony formation (Fig. 4k) of Hep3B and Huh7 cells were remarkably promoted after transfection with the EGFR vector compared with the control vector. In addition, the effects of miR-338-5p mimics were attenuated by the EGFR vector (Fig. 4i–k). These results suggested that targeting of EGFR contributed to the inhibitory effect of miR-338-5p on HCC cell proliferation.

### miR-338-5p inhibits ABCB1 expression by targeting the EGFR/ERK1/2 signaling pathway

**Fig. 5:**
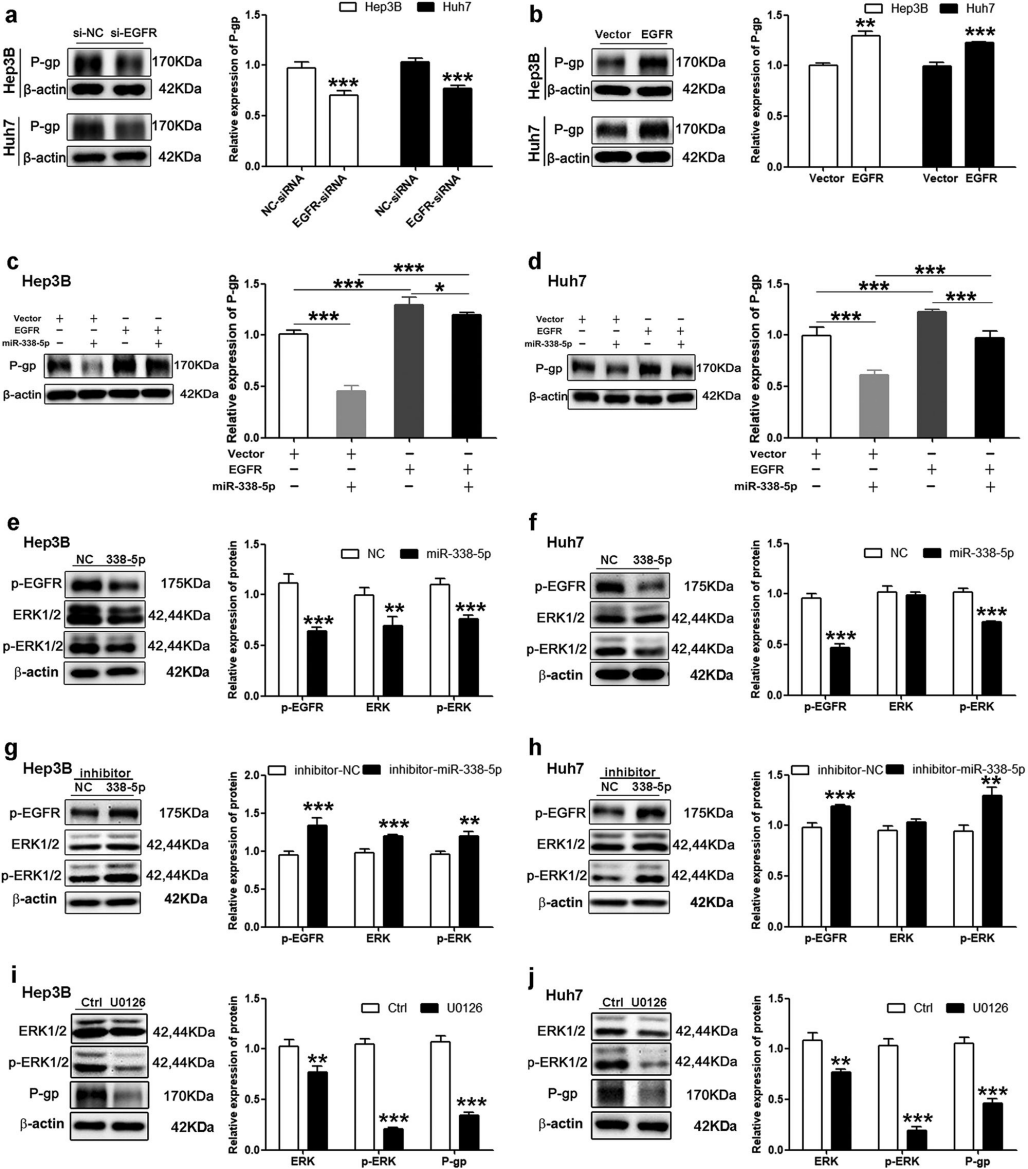
miR-338-5p inhibits ABCB1 expression by targeting the EGFR/ERK1/2 signaling pathway. **a** The expression levels of P-gp were determined by western blotting after transfection with EGFR siRNA. **b** P-gp was increased in cells treated with the EGFR vector. **c**–**d** The P-gp levels in cells after co-transfection with miR-338-5p and EGFR vector. Western blot showing expression levels of p-EGFR, ERK1/2, and p-ERK1/2 in cells transfected with miR-338-5p mimics **e**–**f** or inhibitors **g**–**h**. **i**–**j** HCC cells were treated with 15 μM U0126 (an inhibitor of ERK1/2 signaling) for 24 h, and then, the protein levels of genes were detected by western blot analysis. Student’s *t*-test (two-tailed) or one-way analysis of variance was employed to analyze the data. Data in all experiments are presented as the means ± SD of three independent experiments. **P < 0.01, ***P < 0.001 vs. NC, Vector or Ctrl

In the present study (Fig. 1e, f), the inhibitory effect of miR-338-5p on P-gp expression was still obvious after transfection with ABCB1 (cds), which indicated that miR-338-5p might reduce exogenous P-gp via a non-classic regulatory mechanism of miRNA. Given that the expression of ABCB1 can be regulated by the EGFR signaling pathway^29,30^ and EGFR was discovered as another target of miR-338-5p, the association among EGFR, P-gp and miR-338-5p was examined. First, we observed that silencing or overexpressing EGFR, respectively, decreased or increased the expression of ABCB1 mRNA (Figure S4a and b) and protein (Fig. 5a, b) in both Hep3B and Huh7 cells. Furthermore, the inhibitory effects of miR-338-5p on P-gp expression were tested under the condition of EGFR overexpression. As shown in Fig. 5c, d, the effects of miR-338-5p on P-gp were significantly reversed by the overexpression of EGFR.

To determine whether EGFR-mediated signaling participated in the inhibitory effect of miR-338-5p on ABCB1, we examined the levels of the major components of the EGFR pathway, including p-EGFR, ERK1/2, p-ERK1/2, AKT, p-AKT, JNK, p-JNK, P38, and p-P38, in the HCC cells overexpressing miR-338-5p. The results showed that miR-338-5p overexpression inhibited the levels of phosphorylated EGFR and ERK1/2 (Fig. 5e, f) but not those of AKT, JNK, and P38 (Figure S4c) in Hep3B and Huh7 cells. In addition, the levels of phosphorylated EGFR and ERK1/2 were enhanced in HCC cells after silencing miR-338-5p (Fig. 5g, h). Moreover, we further verified whether ERK1/2 regulated the expression of ABCB1 in HCC cells. The cells were treated with U0126 (an inhibitor of ERK1/2 signaling) for 24 h, and then, the P-gp levels were detected. As shown in Fig. 5i, j, the inhibition of ERK1/2 phosphorylation obviously repressed P-gp expression in HCC cells. The suppressive effects of EGFR siRNA on the expression of ERK1/2 and p-ERK1/2 were confirmed by western blot analysis in HCC cells (Figure S4d and e). Furthermore, inhibition of ERK1/2 obviously increased the sensitivity of HCC cells to DOX (Figure S4f and g). These results demonstrated that in addition to directly binding to the 3′-UTR of ABCB1, miR-338-5p also regulated P-gp expression via the EGFR/ERK1/2 signaling pathway. Together, these data revealed the direct (targeting of ABCB1 3′UTR) and indirect (via the EGFR/ERK1/2-dependent signaling pathway) regulation of ABCB1 by miR-338-5p.

### The miR-338-5p level is negatively correlated with ABCB1 and EGFR mRNA levels in HCC clinical samples

**Fig. 6:**
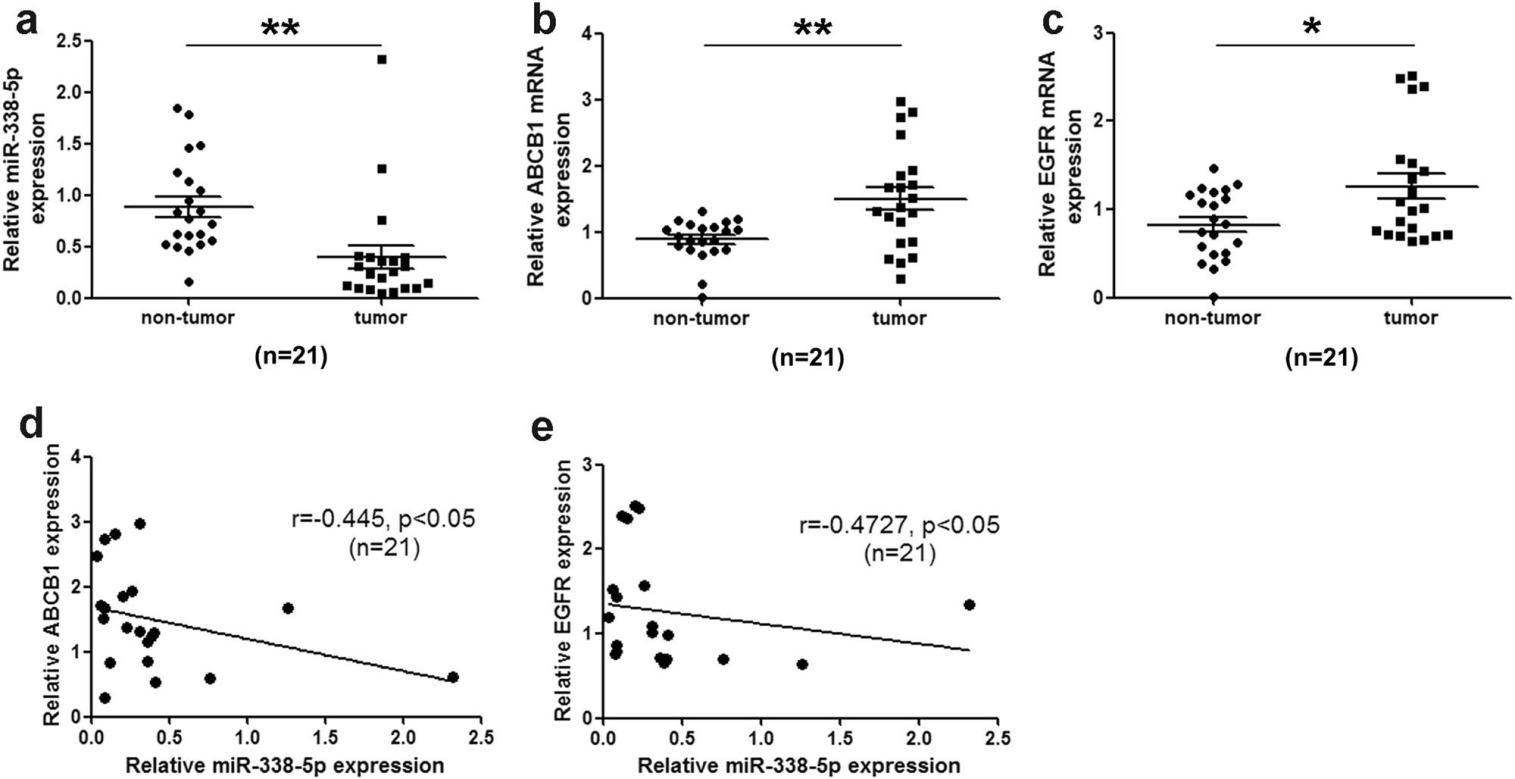
The miR-338-5p level is negatively correlated with the ABCB1 and EGFR mRNA levels in HCC clinical samples. **a**–**c** The expression of miR-338-5p, ABCB1, and EGFR were detected by qRT–PCR. **d** The expression of ABCB1 mRNA was inversely associated with the miR-338-5p expression in the HCC tissues. **e** The correlation between the expression of EGFR mRNA and miR-338-5p in the HCC tissues. Student’s *t*-test (two-tailed) or Spearman’s correlation test was used to analyze the data. Data in all experiments are presented as the means ± SD of three independent experiments. *P < 0.05, **P < 0.01 vs. non-tumor tissues

We further investigated the correlation between miR-338-5p and ABCB1 or EGFR in HCC to evaluate the potential clinical application of miR-338-5p. The levels of miR-338-5p, ABCB1, and EGFR were determined by qRT–PCR in 21 pairs of HCC samples. Compared with matched non-tumor clinical specimens, miR-338-5p was remarkably decreased in liver tumors (Fig. 6a). Moreover, ABCB1 (Fig. 6b) and EGFR (Fig. 6c) were obviously upregulated in HCC samples. Spearman’s correlation analysis revealed that the expression of miR-338-5p was inversely correlated with that of ABCB1 (correlation coefficient *r* = −0.445, *p* < 0.05; Fig. 6d) or EGFR (correlation coefficient *r *= −0.4727, *p* < 0.05; Fig. 6e).

## Discussion

HCC is a primary lethal neoplasm of the liver and one of the most resistant cancers to chemotherapeutic drugs, partially related to the overexpression of ABC transporters.^31^ P-gp was the best characterized member of ABC transporters and could contribute to MDR when its expression or function changed. Recent studies have shown that miRNAs play a critical role in the MDR of HCC and could modulate MDR by regulating P-gp. miR-451 sensitizes breast cancer cells to doxorubicin via targeting ABCB1.^32^ Wu et al. found that the methylation of miR-129-5p CpG islands could regulate multidrug resistance in gastric cancer by targeting ABC transporters.^33^ Let-7 modulates the acquired resistance of ovarian cancer to taxanes via the IMP-1-mediated stabilization of ABCB1.^34^

In the present study, we demonstrated that miR-338-5p sensitizes HCC cells to DOX and VBL by targeting ABCB1. The overexpression or knockdown of miR-338-5p, respectively, repressed or increased the mRNA and protein levels of ABCB1 in Hep3B and Huh7 cells compared with their controls, and the inhibitory effects of miR-338-5p on ABCB1 expression were reversed by the overexpression of ABCB1. These data suggested that ABCB1 was a target of miR-338-5p, and the luciferase assay confirmed that miR-338-5p regulated ABCB1 by directly binding to the 3′-UTR. Furthermore, P-gp-mediated MDR functional assays revealed that miR-338-5p could markedly enhance the response of HCC cells to chemotherapeutic drugs and inhibit the function of P-gp.

Although several reports have demonstrated that miR-338-5p regulates the tumor growth of cancers^27,28^, the role of miR-338-5p in HCC progression needs further illumination. The results of the present study showed that the ectopic expression of miR-338-5p significantly suppressed the cell growth and colony formation of Hep3B and Huh7 cells. In contrast, miR-338-5p inhibitors enhanced the proliferation and colony formation of Hep3B and Huh7 cells. Moreover, silencing of P-gp with siRNA had no effect on the proliferation and colony formation of HCC cells. These data indicated that there might be other targets that mediate the inhibition of tumor cell growth by miR-338-5p. We also predicted the potential targets of miR-338-5p using bioinformatics algorithms and found that epidermal growth factor receptor (EGFR) was another target of miR-338-5p. EGFR belongs to the HER family of tyrosine kinase, which is widely distributed on the surface of mammalian cell membranes.^35^ EGFR plays an important role in the etiology and progression of many carcinomas, including HCC.^36,37^ Here we found that the proliferation and colony formation of Hep3B and Huh7 cells were markedly inhibited when EGFR was silenced, and the suppressive tumorigenic property of miR-338-5p was mostly restored when EGFR was overexpressed in these HCC cells. Although the partial recovery of the miR-338-5p inhibitory function in cell proliferation could be explained by the phenomena that miR-338-5p still inhibited EGFR protein levels after overexpressing EGFR, we could not rule out the potential existence of other underlying mechanisms; thus, further investigation is needed in the future. Taken together, these results suggested that miR-338-5p suppressed the proliferation of HCC cells at least in part by targeting EGFR.

**Fig. 7:**
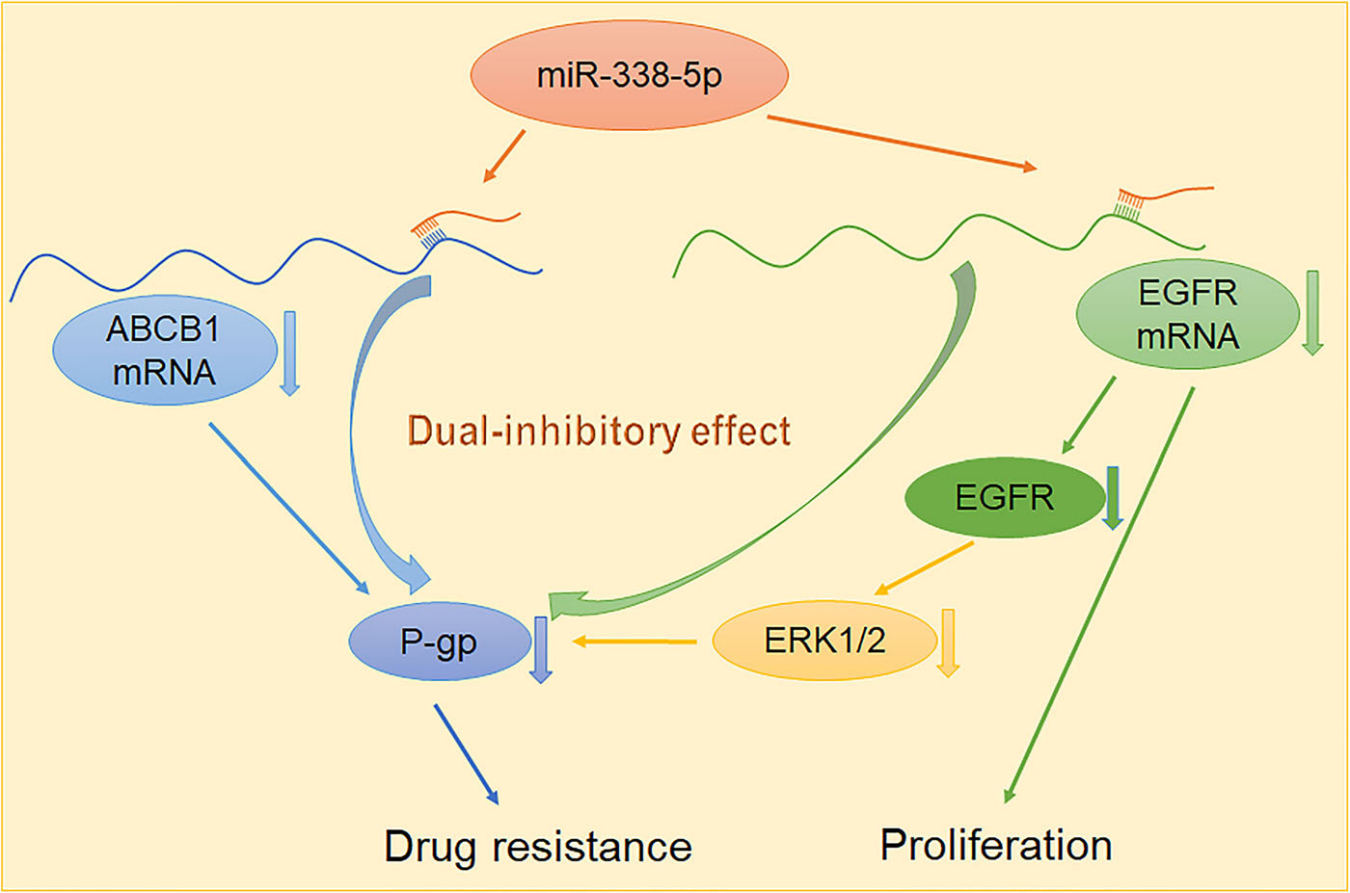
Schematic model depicting the miR-338-5p/EGFR/ABCB1 regulatory loop in the drug resistance and proliferation of HCC

Previous studies have indicated that EGFR signaling is also involved in the regulation of ABCB1 expression.^38,39^ Hence, we determined whether the EGFR signaling cascade was the target of miR-338-5p in the regulation of P-gp. ABCB1 was markedly decreased or increased at the protein level when EGFR was downregulated or enhanced, respectively, and the inhibitory effects of miR-338-5p on the expression of P-gp were significantly reversed by the overexpression of EGFR. Notably, the overexpression of miR-338-5p inhibited the level of phosphorylated EGFR and ERK1/2, and P-gp expression was suppressed after the inhibition of ERK1/2 phosphorylation in HCC cells. Moreover, the miR-338-5p level was negatively correlated with ABCB1 and EGFR mRNA levels in 21 HCC clinical samples. Although this result contradicted that of Chen et al.,^40^ the main limitation of both studies was the small number of HCC samples; thus, further validation of the role of miR-338-5p in more patients is needed in the future. According to Liang et al.,^41^ EGFR inhibits the expression of miR-338-3p via HIF-1α binding to the miR-338-3p promoter in breast cancer. In the present study, we determined whether EGFR could regulate the expression of miR-338-5p in HCC. As shown in Figure S4h and i, EGFR had no effect on miR-338-5p expression in Hep3B and Huh7 cells. The different types of cancers and miRNA isoforms used by Liang and in the present study may explain these phenomena. Furthermore, the present results revealed the direct and indirect regulation of ABCB1 by miR-338-5p (Fig. 7). On the one hand, ectopic miR-338-5p expression decreased ABCB1 expression by directly targeting its 3′-UTR. On the other hand, the inhibition of the EGFR/ERK1/2 signaling pathway by miR-338-5p via direct targeting of the 3′-UTR of EGFR suppressed ABCB1 expression.

In conclusion, the present study demonstrated the critical roles of miR-338-5p in the MDR and growth of HCC by targeting ABCB1 and EGFR. Considering that the present results revealed attenuated miR-338-5p expression and enhanced ABCB1 and EGFR expression in human clinical HCC specimens, the novel identified miR-338-5p/EGFR/ABCB1 axis may provide new insight into the mechanisms underlying MDR and the pathogenesis of HCC, and the restoration of miR-338-5p expression may be a potential therapeutic strategy for the treatment of HCC in the future.

## Electronic supplementary material

Supplementary Material
