# Supplementary material for: The dual-inhibitory effect of miR-338-5p on the multidrug resistance and cell growth of hepatocellular carcinoma
Source: Signal Transduct Target Ther. 2018 Jan 26;3:3. doi: 10.1038/s41392-017-0003-4 (PMC5837112; doi:10.1038/s41392-017-0003-4)
Supplement: Supplementary file 1 — Supplementary Material [file 41392_2017_3_MOESM1_ESM.docx]

**The dual-inhibitory effect of miR-338-5p on multidrug resistance and cell growth of hepatocellular carcinoma**

Yang Zhao^1, 2^, Jing Chen^1, 2^, Wenxin Wei^3^, Xinming Qi^1, 2^, Chunzhu Li^1, 2, **^, Jin Ren^1, 2,*^

*^1^**Center for Drug Safety Evaluation and Research, State Key Laboratory of Drug Research,* *Shanghai Institute of Materia Medica, Chinese Academy of Sciences, Shanghai 201203, China;*

*^2^Center for Drug Safety Evaluation and Research, State Key Laboratory of Drug Research, Shanghai Institute of Materia Medica, University of Chinese Academy of Sciences, Beijing 100049, China;*

*^3^Department of Hepatic Surgery, Eastern Hepatobiliary Surgery Hospital, Second Military Medical University, Shanghai200438, China*

* Corresponding author. Center for Drug Safety Evaluation and Research, Shanghai Institute of Materia Medica, 501 Haike Road, Shanghai 201203, China, Tel: 86(21) +86-21-20231000#1303, Fax: +86-21- 20231000#1303.

** Corresponding author. Center for Drug Safety Evaluation and Research, Shanghai Institute of Materia Medica, 501 Haike Road, Shanghai 201203, China, Tel: 86(21) +86-21-20231000#1303, Fax: +86-21- 20231000#1303.

*E-mail addresses*: czliofficial@126.com (C. Li), jren@cdser.simm.ac.cn (J. Ren).

**Supplementary Information**

This file contains four Supplementary Figures and one Supplementary Table.


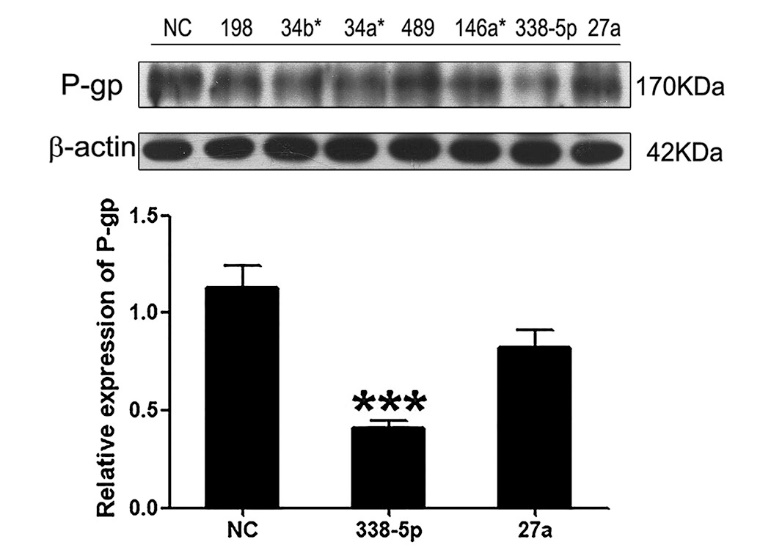


**Figure S1. MiR-338-5p inhibits P-gp expression in human hepatoma cells.** Western blot of the extracts from Hep3B cells transfected with the candidate miRNA mimics. P-gp levels expressed as fold changes relative to values in cells transfected with NC. See the previous study to obtain the statistical results of miR-198, 34b*, 34a* 489 and 146a*.^25^


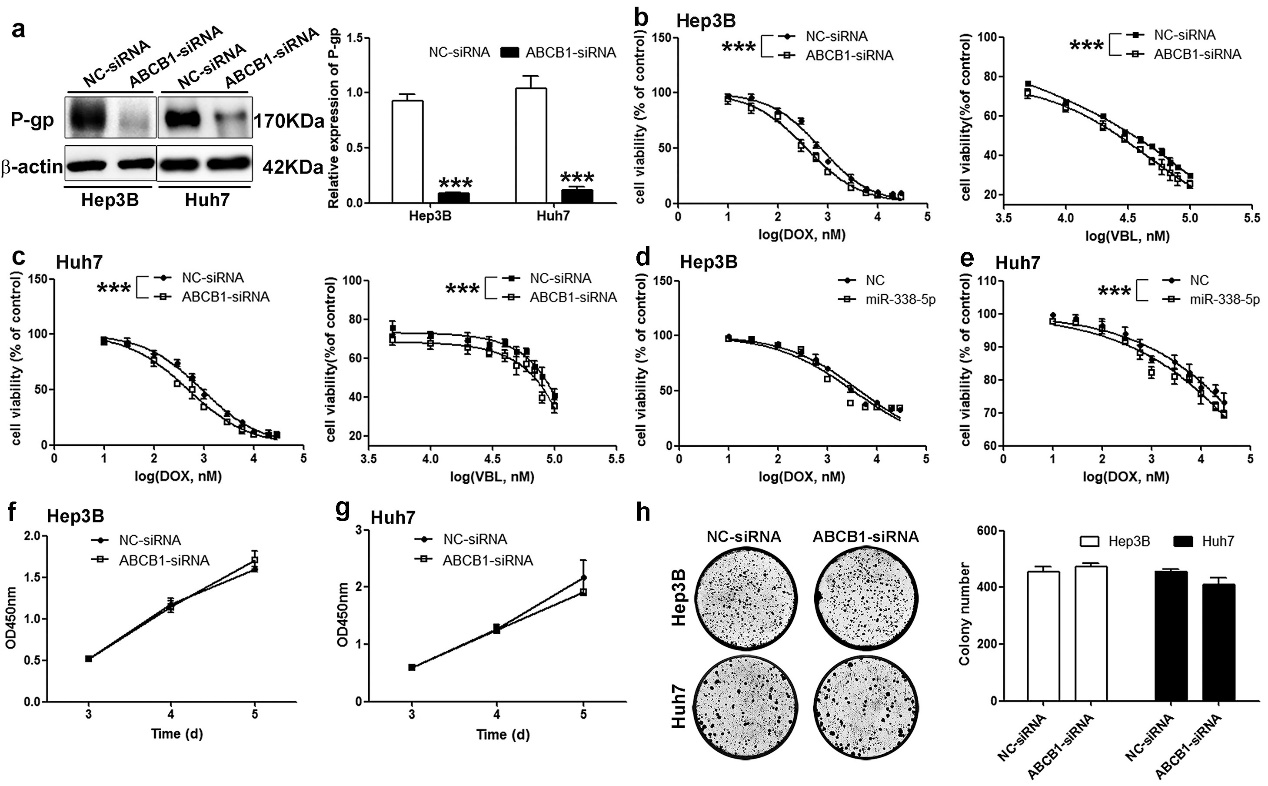


**Figure S2. The effects of silencing ABCB1 on HCC cells drug sensitivity and proliferation.** (a) HCC cells were transfected with NC-siRNA or ABCB1-siRNA for 72h, and then protein was tested by western blot. Cells treated with medium containing DOX or VBL for 48h, and then cell viability was detected. Silencing P-gp increased the sensitivity of Hep3B (b) and Huh7 (c) cells to DOX and VBL. (b-c) Left: The sensitivity of cells to DOX. Right: The sensitivity of cells to VBL. (d-e) Cells treated with medium containing DOX for 24h, and then cell viability was detected. (f-g) Cell proliferation was measured with CCK-8 assay. (h) ABCB1 silencing did not affect the colony formation of cells. ****P*<0.001 vs. NC-siRNA.


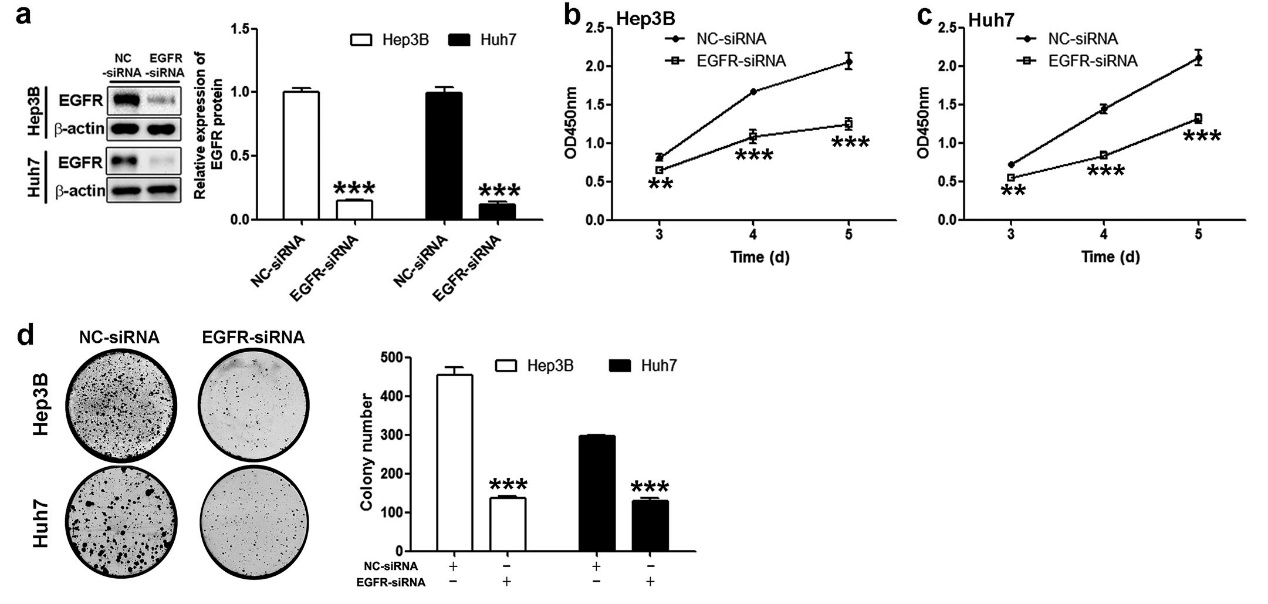


**Figure S3. Silencing EGFR repressed the HCC cells growth.** (a) The effects of siRNAs on EGFR protein expression in HCC cells. The effects of EGFR siRNA on the proliferation (b-c) and colony formation (d) of HCC cells was detected with CCK-8. ***P*<0.01, ****P*<0.001 vs. NC-siRNA.


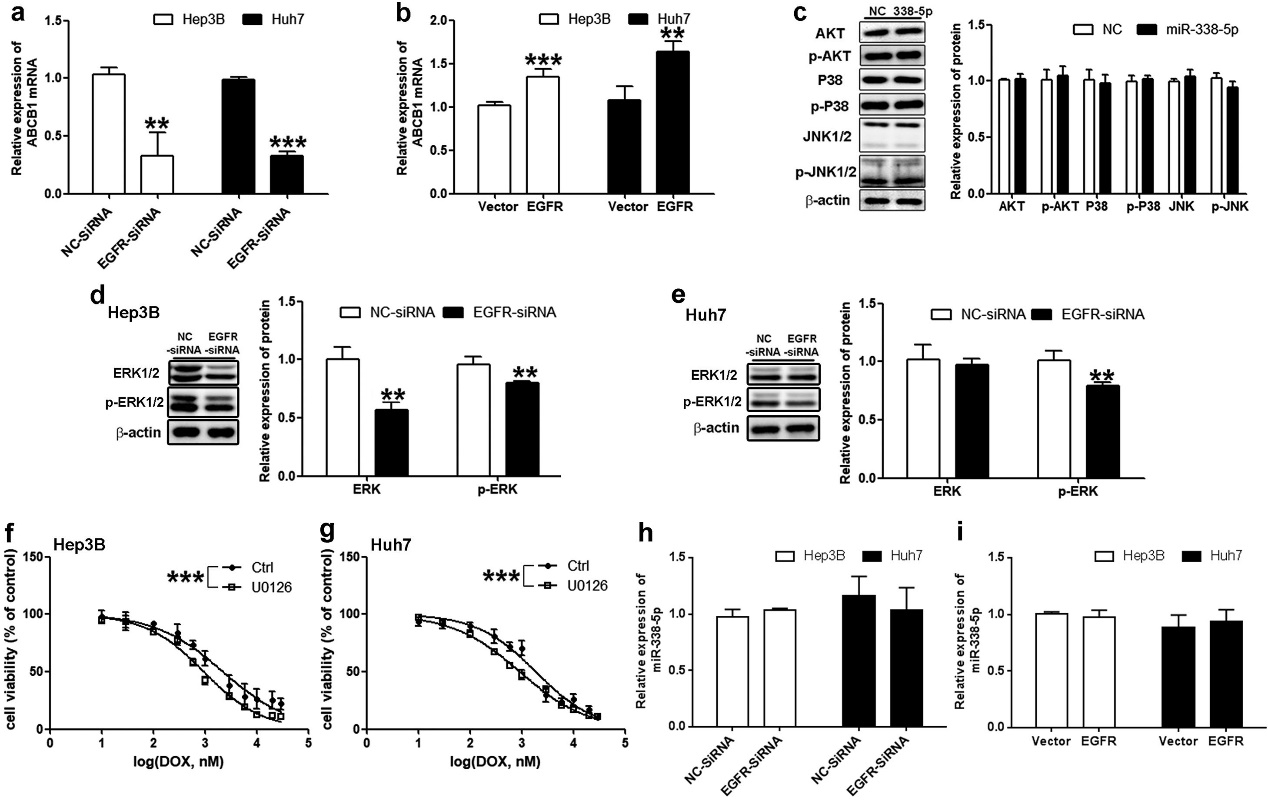


**Figure S4. The protein levels of genes in cells.** (a) The expression levels of ABCB1 mRNA were determined by qRT-PCR after transfected with EGFR siRNA. (b) ABCB1 mRNA was increased in cells after treated with the EGFR vector. (c) Protein levels of genes in Hep3B cells after transfected with miR-338-5p. (d-e) After transfected with EGFR siRNAs for 72h, the protein levels of ERK1/2 and p-ERK1/2 were determined in HCC cells. Inhibiting ERK1/2 increased the sensitivity of Hep3B (f) and Huh7 (g) cells to DOX. The expression levels of miR-338-5p were determined by qRT-PCR after transfected with EGFR-siRNA (h) or EGFR-vector (i). ***P*<0.01, ****P*<0.001 vs. NC-siRNA or Ctrl.

**Table S1.** The sequences of miRNAs mimics, inhibitors and siRNAs of ABCB1 and EGFR.

| **Name** | **Sequence** | |
| --- | --- | --- |
|  | **Sense (5’-3’)** | **Anti-sense (5’-3’)** |
| miR-338-5p mimics  miR-338-5p inhibitor  ABCB1 siRNA  EGFR siRNA | AACAAUAUCCUGGUGCUGAGUG  CACUCAGCACCAGGAUAUUGUU  CACCCAGGCAAUGAUGUAUTT  GCAGUGACUUUCUCAGCAATT | CUCAGCACCAGGAUAUUGUUUU  AUACAUCAUUGCCUGGGUGTT  UUGCUGAGAAAGUCACUGCTT |
